# Catastrophic Antiphospholipid Syndrome Presenting as Bilateral Central Retinal Artery Occlusions

**DOI:** 10.1155/2015/206906

**Published:** 2015-02-02

**Authors:** Steven S. Saraf, Yogin P. Patel, Ankit Desai, Uday R. Desai

**Affiliations:** Department of Ophthalmology, Henry Ford Hospital, Detroit, MI, USA

## Abstract

A previously healthy 22-year-old African American woman presented with bilateral vision loss associated with headache. Her ocular examination was significant for bilateral retinal arterial “boxcarring,” retinal whitening, retinal hemorrhages, and cherry red spots. She was diagnosed with bilateral central retinal artery occlusions and was hospitalized due to concomitant diagnosis of stroke and hypercoagulable state. She was also found to be in heart failure and kidney failure. Rheumatology was consulted and she was diagnosed with catastrophic antiphospholipid syndrome in association with systemic lupus erythematosus. Approximately 7 months after presentation, the patient's vision improved and remained stable at 20/200 and 20/80.

## 1. Introduction

Catastrophic antiphospholipid syndrome (CAPS) is a rare autoimmune entity consisting of life threatening intravascular thrombosis leading to multiorgan failure. Immune complex formation results in widespread platelet aggregation, coagulation, and immune response. The cascade of events is often triggered by a preceding insult such as infection, surgery, or medication change. The disease may present as a primary condition or in association with an underlying process, most commonly systemic lupus erythematosus (SLE). Specific diagnostic criteria include the involvement of at least three organ systems, evidence of intravascular thrombosis, and presence of antiphospholipid antibodies [1].

Ocular involvement is rare, with only 5.8% of cases reporting retinal findings [2]. To our knowledge, there has been one case report in the literature describing CAPS associated with choroidal thrombosis with ultimately good visual prognosis [3]. We present a rare case of CAPS in a previously healthy 22-year-old African American woman presenting with bilateral CRAO with profound vision loss.

## 2. Case Report

A previously healthy 22-year-old African American woman presented with progressive vision loss and headache. She denied any trauma, pain, pain with eye movements, flashes, or floaters but complained of lethargy. A head CT showed a subacute infarct of the posteroparietal and occipital lobes. The patient was hospitalized and a hypercoagulability work-up was ordered. Initial laboratory testing showed anemia, thrombocytopenia, elevated creatinine, and positive antiphospholipid antibodies. Echocardiography revealed valvular vegetations.

**Figure 1:**
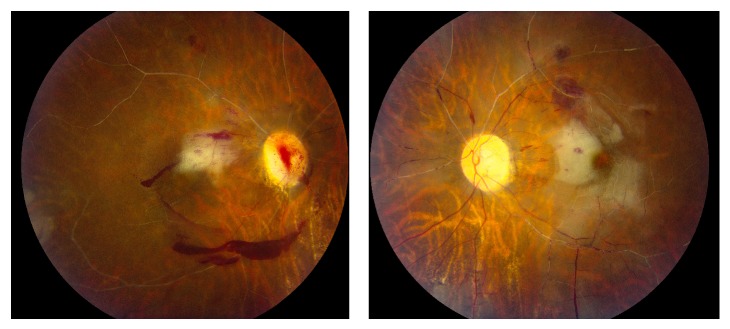
Color fundus photos of each eye show bilateral retinal arterial “boxcarring,” retinal whitening, intraretinal and preretinal hemorrhages, and cherry red spots.

Ophthalmic evaluation demonstrated best corrected visual acuity of hand motions in the right eye and 20/200 in the left eye. An afferent pupillary defect was appreciated in the right eye. Extraocular movements were full. Confrontational visual fields showed global depression in the right eye and inferior hemifield depression in the left. Tonometry was 15 and 11 by applanation in the right and left eyes, respectively. Slit-lamp examination of the right eye was remarkable for anterior vitreous red cells while the left eye was quiet. Dilated fundus exam revealed pale optic nerves, disc hemorrhages, disc neovascularization, arterial attenuation, and arterial “boxcarring.” Multiple retinal hemorrhages, retinal whitening, and a cherry red spot were noted in both eyes (Figure 1).

**Figure 2:**
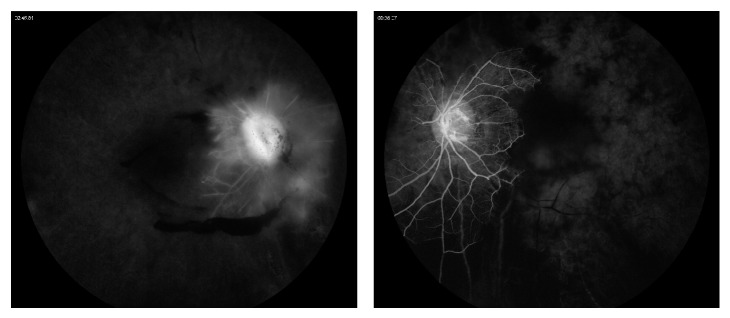
Fluorescein angiography of both eyes in the late phases shows minimal perfusion to the peripapillary areas with late leakage of the filling vessels.

**Figure 3:**
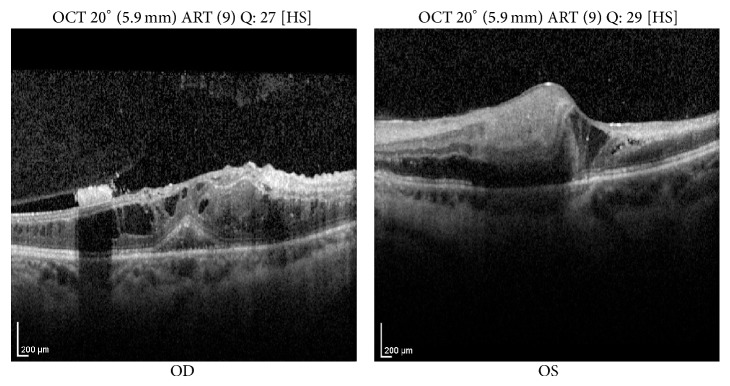
Optical coherence tomography of maculae shows cystoid macular edema and loss of foveal contour.

Fluorescein angiography showed extensive retinal nonperfusion bilaterally (Figure 2). Macular optical coherence tomography showed bilateral cystoid macular edema with distortion of the foveal contour (Figure 3).

Review of systems revealed a recent 30-pound weight gain, difficulty focusing, hair loss, skin changes, and constipation. Rheumatological work-up was significant for positive ANA, anti-Smith antibodies, cardiolipin antibodies, lupus anticoagulant test, and RPR. Given the clinical presentation, laboratory work-up, and imaging, the patient was diagnosed with SLE, antiphospholipid antibody syndrome, and catastrophic antiphospholipid syndrome (Tables 1, 2, and 3).

The patient was given IV methylprednisolone followed by an oral prednisone taper. She was later initiated on mycophenolate mofetil. She underwent panretinal photocoagulation. Her kidney failure recovered while in the hospital and she later required a mitral valve replacement.

## 3. Discussion

Though there can be overlap, catastrophic antiphospholipid syndrome is a distinct entity from primary antiphospholipid syndrome (APS) and systemic lupus erythematosus. CAPS was first described by Asherson in 1992 as a multiorgan vasoocclusive crisis characterized by small vessel thromboses. By definition, it must involve at least three organ systems accompanied by high levels of cardiolipin antibodies or lupus anticoagulant (Table 3). Often seen, though not necessary for diagnosis, are thrombocytopenia and microangiopathic hemolytic anemia [5]. CAPS differs from anti-phospholipid antibody syndrome due to its acute nature and the size of vessels involved. In addition, CAPS can lead to an acute episode of widespread small vessel thrombosis, significant morbidity, and treatment in the ICU setting.

The CAPS registry shows that the most commonly affected organ systems include the kidneys (73%), lungs (59%), brain (56%), heart (50%), and skin (45%). Retinal involvement is relatively uncommon and has been reported in 5.8% of cases. There is a 39% mortality rate with causes of death commonly involving large vessel occlusions in the brain and acute respiratory distress syndrome. Patients are often conscious at the time of presentation but may rapidly deteriorate and become comatose [2].

The etiology of CAPS is primarily speculative as it is a difficult disease to study given its rarity and its likely underdiagnosis in the ICU setting. One theory is the “two-hit hypothesis” where the patient has a known underlying predisposition including HLA genotype or other prothrombotic conditions such as malignancy, vasculitis, SLE, or other rheumatologic conditions [6]. The “second hit” can then be identified in approximately 50% of cases. This may include infection (22%), trauma or surgery (13%), anticoagulation withdrawal (7.2%), neoplasia (6.8%), and lupus flares (3%) [2]. In the case of our patient, no inciting trigger was identified.

An alternate theory is the “thrombotic storm hypothesis” described by Kitchens, who postulated that patients with preexisting hypercoagulability may spontaneously form a thrombus leading to an overall increase in systemic thrombogenic factors, depletion of natural anticoagulant proteins, and an increase in plasminogen activator inhibitors. This thrombotic milieu becomes self-perpetuating and leads to widespread small vessel thrombosis [7].

A third theory states that infections may induce thrombotic activity via molecular mimicry. Infection leads to a transient increase in circulating antiphospholipid antibodies from activated B cells. T cells are directly activated by toxins released by bacteria such as* Staphylococcus* and* Enterococcus* species leading to further stimulation of antibody production and eventual widespread thrombosis.

The primary focus of treatment is to suppress thrombotic activity and immunologic contributions to the prothrombotic state. Treatment usually begins with intravenous anticoagulation with transition to warfarin once a therapeutic INR is reached. Traditionally, patients with APS with venous thrombosis are maintained at an INR of 2-3, but arterial or recurrent thrombosis requires an INR >3 [8]. No definite criteria exist for CAPS, but our patient has been maintained at INR 2-3 without recurrence. Long term immunosuppression can be achieved by intravenous immunoglobulin (IVIG), corticosteroids, or immunologic agents such as mycophenolate mofetil and cyclophosphamide [2]. Our case highlights the importance of interdisciplinary care with internal medicine, rheumatology, and cardiology.

To our knowledge, there has been one other case report describing profound retinal changes related to CAPS. Her ocular symptoms resolved entirely after systemic treatment with IVIG and corticosteroids [3]. In contrast, our patient was thought to require intervention with panretinal photocoagulation due to the profound degree of retinal ischemia, in addition to corticosteroids and immune modulators. However, there are no established guidelines on the management of retinal vasoocclusive disease related to CAPS. Further attention and study are warranted to understand the consequences of CAPS in the visual system and elucidate the need for intervention by the ophthalmologist.

## Figures and Tables

**Table 1:** Revised diagnostic criteria for systemic lupus erythematosus as defined by SLICC.

SLICC diagnostic criteria for systemic lupus erythematosus [4]	

Requirements include at least 4 criteria	
At least one criterion must be met in the clinical and laboratory categories	

Clinical criteria	Laboratory criteria

Acute cutaneous lupus	ANA
Chronic cutaneous lupus	Anti-DNA
Oral or nasal ulcers	Anti-Sm
Nonscarring alopecia	Antiphospholipid antibodies
Arthritis	Low complement (C3, C4, and CH50)
Serositis	Direct Coombs test
Renal^*^	
Neurologic^‡^	
Hemolytic anemia	
Leukopenia	
Thrombocytopenia	

^*^High urine protein-to-creatinine ratio or red blood cell casts.

^‡^Seizures, psychosis, mononeuritis multiplex, myelitis, peripheral or cranial neuropathy, and acute confusional state, in the absence of other explainable causes.

**Table 2:** Sapporo criteria for diagnosis of antiphospholipid antibody syndrome.

Diagnosis of antiphospholipid antibody syndrome [9]	

Requirements include at least one clinical and one laboratory criterion In the absence of an underlying cause, the diagnosis of primary antiphospholipid syndrome is made In the presence of SLE, the diagnosis of secondary antiphospholipid syndrome is made	

*Clinical criteria *	
Vascular thrombosis	
One or more episodes of venous, arterial, or small vessel thrombosis without evidence of thrombosis in surrounding tissues	
Pregnancy morbidity	
Unexplained fetal death at >10 weeks of gestation in an otherwise normal fetus	
One or more premature births before 34 weeks of gestation due to eclampsia, preeclampsia, or placental insufficiency in a morphologically normal neonate	
Three or more consecutive unexplained <10-week pregnancy losses	

*Laboratory criteria *	
IgG or IgM anticardiolipin antibodies	
Greater than 40 GPL or MPL units or >99th percentile for the testing laboratory	
Detected on two or more occasions at least six weeks apart	
IgG or IgM beta 2-glycoprotein I antibodies	
Titer greater than 99th percentile for the testing laboratory	
Lupus anticoagulant activity	
Positive study is based on guidelines established by Scientific Standardization Committee (SCC) [10]	
Detected on two or more occasions at least 6 weeks apart	

**Table 3:** Diagnostic criteria for diagnosis of catastrophic antiphospholipid syndrome as defined by International Congress on Antiphospholipid Antibodies Task Force.

Diagnostic criteria for catastrophic antiphospholipid syndrome (CAPS) [2]	

All four criteria must be met for definite diagnosis of CAPS	
Probable diagnosis of CAPS includes three criteria	

*Diagnostic criteria *	
(1) Evidence of involvement of 3 organs, systems, and/or tissues	
(2) Development of manifestations within a 1-week span	
(3) Presence of antiphospholipid antibodies	
Lupus anticoagulant (positive study is based on guidelines established by SCC)	
Anticardiolipin antibodies (in titers higher than 40 GPL)	
Beta 2-glycoprotein I antibodies (in titers higher than 40 GPL)	
(4) Findings unexplained by other diagnoses	

^*^Scientific Standardization Committee [10].
